# Mutual associations between intellectual disability and epilepsy-related psychiatry disability: Population-based study

**DOI:** 10.1097/MD.0000000000006831

**Published:** 2017-05-12

**Authors:** Zhenjie Wang, Chao Guo, Gong Chen, Lei Zhang, Xun Wen, Xiaoying Zheng

**Affiliations:** Institute of Population Research/WHO Collaborating Center on Reproductive Health and Population Science, Peking University, Beijing, China.

**Keywords:** China, epilepsy, intellectual disability, psychiatry disability

## Abstract

Epilepsy is the third-leading cause of psychiatry disability in China, and intellectual disability (ID) is also 1 major type of disabilities in China. This study estimates the prevalence of comorbidities with ID and epilepsy-related psychiatry disability (EPD) and examines mutual associations within ID and EPD.

Data were taken from the Second China National Sample Survey on Disability, which was a nationally representative, population-based survey. To derive a nationally representative sample, the survey used multistage, stratified, cluster random sampling with probability proportional to size. The disabled people who had ID and EPD based on the World Health Organization International Classification of Functioning, Disability, and Health and the International Statistical Classification of Diseases. The cox-proportional hazards model was used to examine the associations between ID and EPD considering the happened sequence of ID and EPD.

The prevalence of ID with EPD was 0.14 (95% confidence interval: 0.09–0.19) per 1000 people. Age was strongly associated with the risk of EPD, which was diagnosed after ID, especially among young ID population. Except for age, other variables were also associated between ID and EPD considering sequence of ID and EPD.

This study is the first national study to explore mutual associations with ID and EPD and highlights the young ID children with high risk of development of epilepsy. To address the challenge of ID with EPD disability in China, the government should adjust its strategies for healthcare systems to prevent disability.

## 1 Introduction

According to the World Health Organization (WHO)'s estimation, there were 8 out of 1000 people, who were suffered by epilepsy.^[1]^ A number of people with epilepsy might also be effected by other health conditions,^[2,3]^ such as psychiatric disorders,^[4–7]^ somatic comorbidities,^[2,8,9]^ or intellectual disorders.^[10]^ In China, epilepsy has been reported as the third-leading cause of psychiatry disability.^[11]^ Comparing with the general population, people with epilepsy nearly have 3 to 4 times risk for premature death.^[12]^ Comorbidities of epilepsy with other health conditions are always associated with healthcare needs, quality of life, and mortality.^[2,3]^

Intellectual disability (ID) refers to lower than normal intellectual ability and is accompanied by adaptive behavior disorders. This kind of disability results from impairment of the structure and functions of the nervous system, limits individual activity and participation, and requires all-round, extensive, limited, or intermittent support.^[11]^ Previous articles suggested the prevalence of ID varies widely, it has been estimated that approximately 2% of the adult population have ID.^[13,14]^

Comorbidities of ID and epilepsy may be a common combination of diseases. Over 50% of a representative sample with ID and active epilepsy were reported to have various psychiatric diagnoses.^[10]^ Epilepsy-related psychiatry disability (EPD) was a serious performance of epilepsy, and was easily diagnosed in clinical researches. A previous study reported comorbidities of EPD with psychiatric disorders, such as organic mental disorders, dementia, and so on in China.^[15]^ Although EPD is related with other mental disorders and epilepsy disease is more common in people with ID than in the general population, there is no a nationwide population-based survey on EPD with ID reported in China. In the study here, we used data from a nationwide survey on disabilities to assess the mutual associations between EPD and ID.^[16]^

## 2 Methods

### 2.1 Data source

In the present study, we used the Second China National Sample Survey on Disability, which was a nationally representative sample. The survey employed a multistage, stratified random cluster sampling scheme, with probability proportional to size to derive a representative sample. The survey protocol and questions were reviewed by leading national and international experts.^[16]^ The sampling scheme of this survey was reviewed by experts from the Division of Statistics of the United Nations.^[16]^ This survey was conducted from April 1 to May 31, 2006. The survey covered all provincial administrative areas in mainland China, excluding Hong Kong, Macau, and Chinese Taipei. Details of the survey design were described elsewhere.^[11]^

### 2.2 Ethics

The surveys were approved by the State Council (Guo Ban Fa No 73 [2004]). The survey was conducted within the legal framework governed by statistical law in China. All survey respondents provided consent to participate in these surveys and clinical diagnosis.

### 2.3 Data collection procedures and data quality

Before the formal survey, a pilot study was conducted.^[16]^ During data collection, strict quality control measures were implemented at every step, a structured interview questionnaire was used to inquire about disabilities.^[16]^ Subjects who responded “yes” to any of the corresponding questions were assigned to different designated physicians for further disability screening and confirmation. Following the guidelines of diagnostic manuals, designated physicians performed the medical examinations, made a final diagnosis of the disability, if any, then assessed its severity and confirmed the primary cause.^[16]^ Respondents with multiple positive answers were examined by a separate doctor for each disability.

After the field investigations, the teams made a home revisits for conduct surveys in the quarters chosen for postsurvey quality checks and calculate errors in the survey overall. The results of the quality checks showed that the omission rate of the resident population was 1.31 per 1000 persons; the omission rate of the disabled population was 1.12 per 1000 persons.^[16]^

### 2.4 Identification of people with EPD and ID

Psychiatry disability was defined and classified by the expert committee of the Second China National Sample Survey on Disability, based on the WHO International Classification of Functioning, Disability and Health (WHO-ICF).^[17]^ EPD was diagnosed by professional psychiatrists according to the item G40 and G41 of the International Statistical Classification of Diseases, 10th Revision and WHO-ICF.^[17,18]^

ID refers to psychiatry functioning generally lower than that of normal people, accompanied by adaptive behavior disorders. ID was diagnosed by the Gesell Developmental Scales among children aged 0 to 6 years, the Wechsler Intelligence Scale for Children—Chinese Revised among children 7 to 16 years, and the Wechsler Adult Intelligence Scale—Revised Chinese among those aged older 17 years.^[19–21]^ Children aged 0 to 6 years with a development quotient lower than 75 and those aged 7 to 17 years with an intelligence quotient lower than 70 were diagnosed as having ID.^[16]^

All the classifications and grading standards, screening methods, diagnosing methods, and relevant scales of disabilities were pretested in pilot studies, and had good reliability and validity.

### 2.5 Statistical analysis

**Figure 1:**
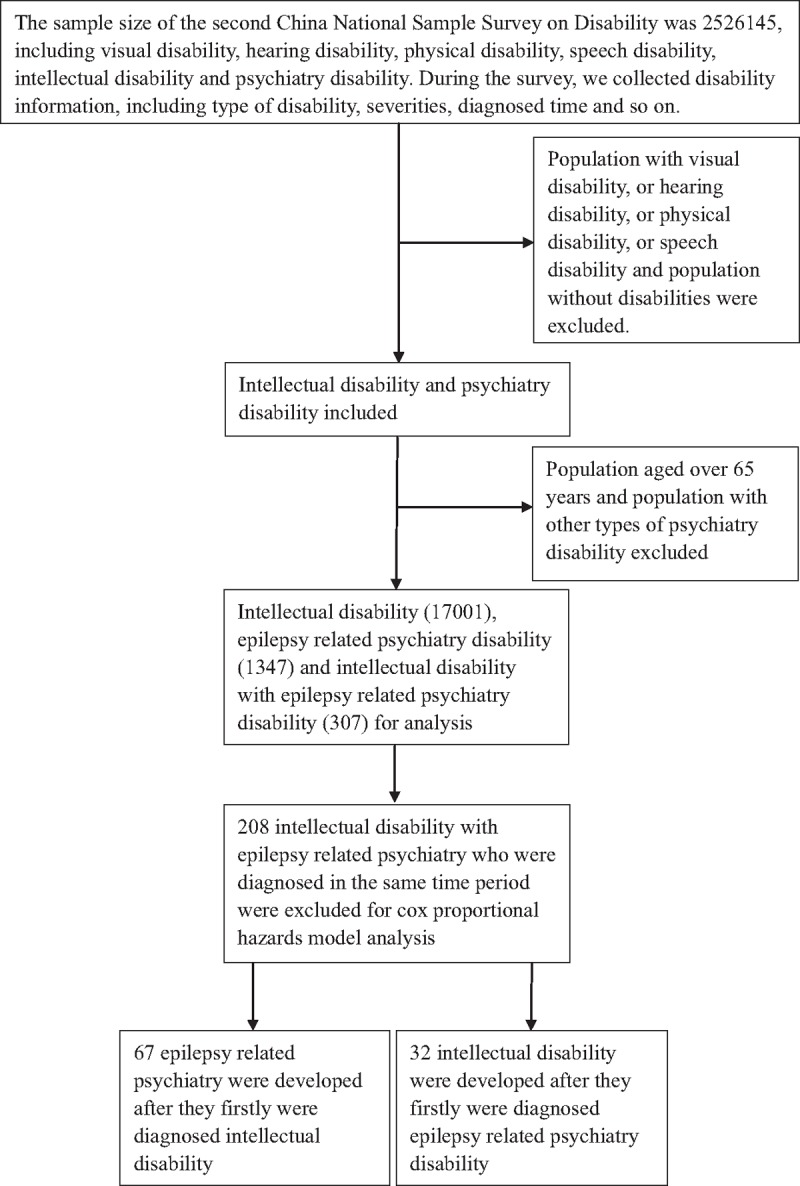
Sample size selecting process for analysis.

The present study collected information on ID diagnosed time before EPD using a binary category (yes or no), EPD diagnosed time before ID using a binary category (yes or no), age groups in 2006 (40–64, 20–39, and 0–19), gender (male or female), residential area (urban or rural: according to “Hu Kou”), ethnicity (Han or other), household size (1–3, 4–6, or 7–9 people), and household income above average in 2006 (yes or no). We used a cox-proportional hazards model to estimate the hazard ratios and 95% confidence interval (CI) for the diagnosed time of ID before EPD and the diagnosed time of EPD before ID for selected variables. Diagnosed time of EPD or ID accorded to subjects’ record during data collection. The sample size selecting steps were summarized in Fig. 1. Statistical significance was set at a 2-tailed *P* value of <.05. Statistical analyses were performed using SAS v. 9.2 (SAS Institute Inc, Cary, NC).

## 3 Results

### 3.1 Characteristics of the subjects

Selected characteristics of the cases of ID with EPD and study subjects are presented in Table 1. The prevalence of ID with EPD was 0.14 (95% CI: 0.09–0.19) per 1000 people. Cases of ID with EPD aged between 10 and 49 accounted for 83.4% of the total number of cases. In the present study, ID with EPD cases or study subjects who were male, resided in rural areas, lived under average income, and were of Han nationality accounted for the majority of sample size.

**Table 1:**
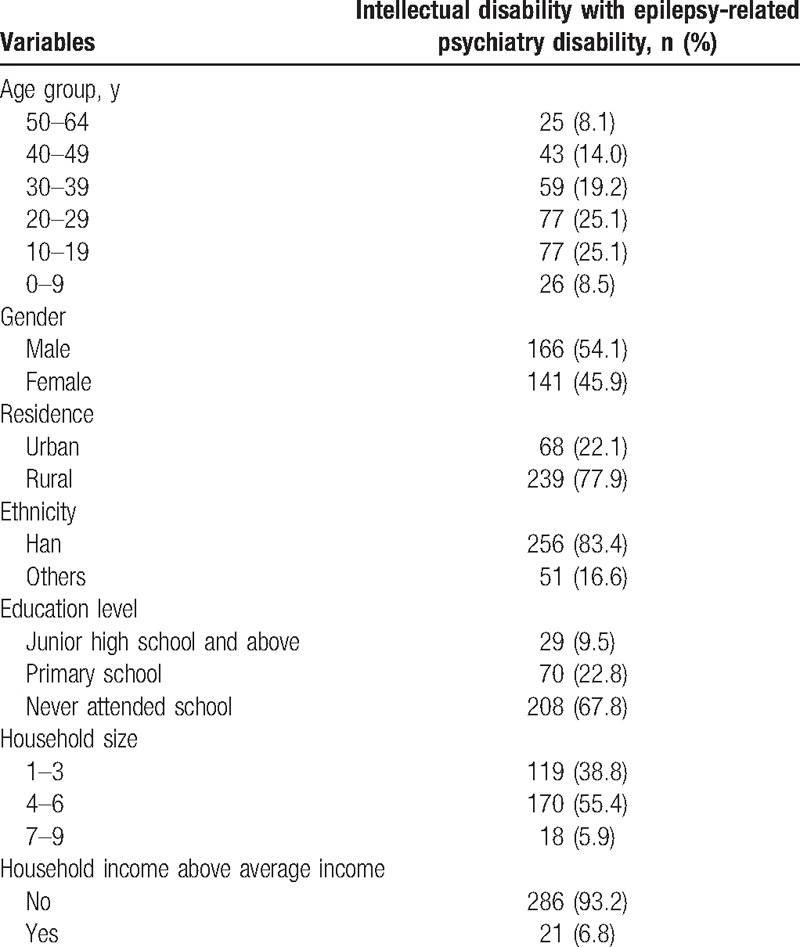
Characteristics of comorbid epilepsy caused psychiatry disability with intellectual disability.

### 3.2 Mutual associations between ID and EPD

Mutual associations between ID and EPD are presented in Tables 2 and 3. Under consideration of the fact that other variables were confounding variables, age was strongly associated with the risk of EPD which was diagnosed after ID, especially for those aged younger than 30. However, we did not observe similar association between age and the risk of EPD's diagnosed time before ID. We also found significant association between household size and the risk of EPD's diagnosed time before ID, considering other variables were confounding variables.

**Table 2:**
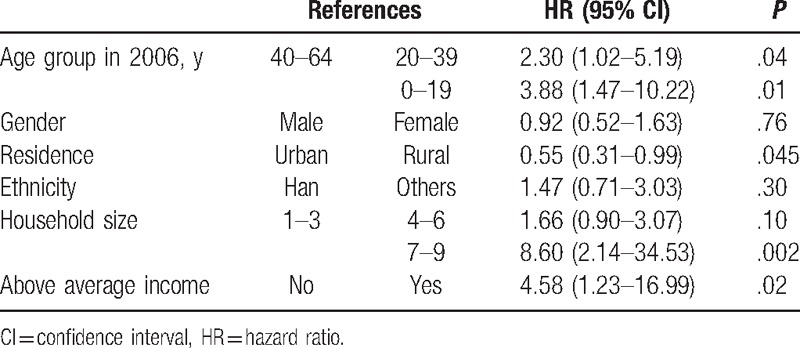
HR (95% CI) of intellectual disability diagnosed time before epilepsy-related psychiatry disability.

**Table 3:**
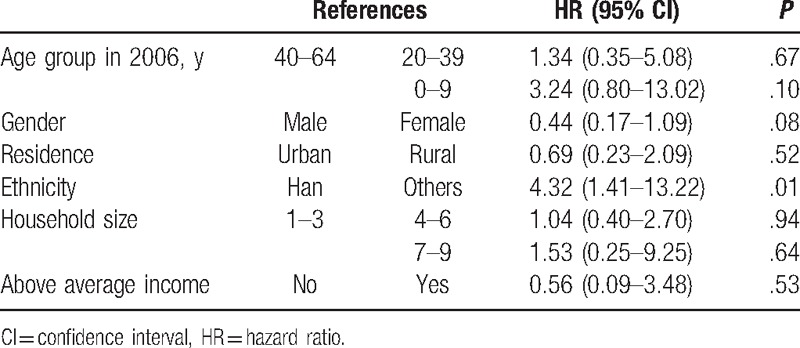
HR (95% CI) of epilepsy caused psychiatry disability diagnosed time before intellectual disability.

## 4 Discussion

### 4.1 Main findings and their significance

The mutual association between ID and EPD has been investigated in China. We used detailed personal interviews and professional examinations of disabilities from the second China National Sample Survey on Disability. We obtained very unique and valuable information on ID with EPD among the Chinese population. The prevalence of ID with EPD was 0.14 per 1000 people. Furthermore, we observed strong mutual association between ID and EPD considering sequence of these disabilities.

### 4.2 Comparison with others studies and implications of the findings

In the study here, we used detailed personal interviews and professional examinations of disabilities from the 2006 nationally representative sample to examine the mutual associations within ID and EPD in China. We obtained valuable results on ID with EPD among the Chinese population. The observed prevalence of ID with EPD was lower than a review indicated.^[10]^ One major reason is that the definition of disabilities in China is narrower than in other countries, which might lead to underestimation of the prevalence of ID with EPD disabilities in China. Moreover, the prevalence of ID with epilepsy might be due to the methods used and inherent population biases, because varied methods used might cause the differences in prevalence estimation.^[22]^ Third, we estimated the prevalence of ID with EPD, did not estimate the prevalence of ID with epilepsy disease. The difference between disease and disability might contribute to this low prevalence. Although the prevalence of ID with EPD was lower than other studies, China was facing a challenge of disabilities. The upward trends in prevalence of disabilities were observed in China.^[11]^ This increased prevalence might have been due to changes in attitudes to disability, increasing public awareness, and changes in diagnostic criteria.^[23]^ Although the awareness about disabilities was improving, the increment in prevalence might also be attributed to the current under-development status of psychiatry health service system in China. Nearly 45% of urban population and 80% of rural population could not access to any type of healthcare insurance in the 2006.^[24]^ In mainland China, the percentage of China's financial expenditures and gross domestic product (approximately 5% in recent years) on healthcare system was much smaller than the percentage in Hong Kong, where the annual government recurrent expenditure on health care increased 40% from 2007 to 2012.^[25]^ Slow development of specialized training, treatment of disability, and culturally rooted stigmas about disability were also barriers to the improvement of health status in Chinese population.

In the present study, we presented more detailed association between age and EPD with ID. Age groups in previous studies were classified as adult, child, or mixed (adult and child)^[10]^ or presented as broad age bands of 0 to 18, 19 to 49, and 50+. The highest prevalence of epilepsy among people with ID was observed among population aged between 19 and 49 years. In our study, the first 3 prevalences of ID with EPD were observed among population aged between 20 and 29 years (0.26 [95% CI: 0.20–0.32] per 1000 people), between 10 and 19 years (0.20 [95% CI: 0.15–0.24] per 1000 people), and between 30 and 39 years (0.14 [95% CI: 0.10–0.17] per 1000 people). The lowest prevalence of ID with EPD was found among population aged 50 and older, which was similar to previous result.^[10]^ Moreover, age was also found as a significant factor for disable sample of population with ID onset before EPD, especially for children. But we did not observe that age was a significant factor among those with EPD onset before ID. Age was not only a demographic variable, but also associated with social roles and social position which came with socioeconomic factors, prestige, and access to resources.^[26]^ Furthermore, normal functioning of children with disability was affected by social participation limits and these children needed more health care. In developed countries, children with epilepsy had less accessed to educational resources^[27,28]^ and presented poorer social skills and sense of control.^[29]^ Under consideration of low development of healthcare or health insurance system in China, the situation was more serious if children had ID and EPD together.

### 4.3 Strengths and limitations

The limitations of this study should be noticed. We did not consider every potential confounder, such as marital status, education, etc., because these factors were consistent with disabled population, which should also be treated with caution for further researches. In addition, the design of this study was an ecological study with all of the limitations on assumptions about causality. The primary strengths of the present study included the large sample size and the representativeness of the sample, which covered all provincial administrative areas in mainland China. In addition, all subjects in the households selected were interviewed face to face at the time of data collection. Also, standardized quality control schemes were in place during the field interviews, the included training of the interviewers, and the cross-checking of returned surveys by contacting survey participants, which resulted in little response bias.

## 5 Conclusion

Currently, China is undergoing social and economic reforms. The current results will benefit our understanding of the prevalence of ID with EPD and risk factors within ID and EPD. Our findings will help policymakers to understand the current status of ID with EPD in China, and also help them to notice the mutual association between ID and EPD. These unique results will be helpful to improve strategies for individuals, communities, and the healthcare/healthcare insurance system to prevent disabilities.
